# In Silico Design of Pyrimidine Derivatives as Potential α-Glucosidase Inhibitors: QSAR, Molecular Docking, ADMET, and Molecular Dynamics Studies

**DOI:** 10.3390/ijms27135696

**Published:** 2026-06-24

**Authors:** Oussama Abchir, Bouchra Rossafi, Amal Bouribab, Bouchra Es-Sounni, Rodouan Touti, Imane Yamari, Abdelouahid Samadi, Samir Chtita

**Affiliations:** 1 Laboratory of Analytical and Molecular Chemistry, Hassan II University of Casablanca, Casablanca 20360, Morocco; oussamaabchir12@gmail.com (O.A.); bouchrarossafi7@gmail.com (B.R.); bouribabamal@gmail.com (A.B.); yamariimane86@gmail.com (I.Y.); 2 Bioorganic Chemistry Team, Faculty of Science, Chouaïb Doukkali University, P.O. Box 24, El Jadida 24000, Morocco; bouchrasounni@gmail.com; 3 Laboratory of Advanced Materials and Applications (LM2A), Faculty of Sciences Dhar El Mahraz, Sidi Mohamed Ben Abdellah University, Fez 30000, Morocco; touti2007@gmail.com; 4 Department of Chemistry, College of Science, United Arab Emirates University, Al Ain P.O. Box 15551, United Arab Emirates

**Keywords:** alpha-glucosidase, diabetes, 2D-QSAR, molecular docking, ADMET, dynamics

## Abstract

Diabetes mellitus remains a major metabolic disorder requiring the development of new and effective α-glucosidase inhibitors. The present study aimed to identify, design, and optimize novel 3-amino-2,4-diarylbenzo[4,5]imidazo[1,2-α]pyrimidine derivatives with promising inhibitory activity against the α-glucosidase enzyme using a comprehensive in silico strategy. Approximately 300 molecular descriptors were calculated to characterize a dataset of 32 compounds (Peytam et al.) and to investigate the structural factors governing their biological activity. Based on these descriptors, a multiple linear regression model was developed to predict the inhibitory activities of the compounds against alpha-glucosidase. The developed model demonstrated satisfactory predictive performance and was internally and externally validated to ensure its accuracy, robustness, and reproducibility. In addition, the applicability domain analysis confirmed the reliability of the predictions. Using the validated QSAR model, seven new derivatives were designed with predicted pIC_50_ values exceeding the maximum activity of the parent compounds. The leverage analysis demonstrated that all newly designed compounds were located within the applicability domain of the model, supporting the reliability of the predictions. To further evaluate their inhibitory potential, molecular docking studies were performed to investigate the interactions between the designed compounds and the α-glucosidase active site. The docking results revealed favorable binding interactions comparable to those reported for known α-glucosidase inhibitors. Furthermore, ADMET analysis indicated generally favorable pharmacokinetic properties, although potential CYP3A4 inhibition-related pharmacokinetic risks were identified and discussed. Molecular dynamics simulations, including replicated runs and MM/GBSA binding free energy calculations, confirmed the stability of the most promising protein–ligand complexes throughout the simulation period. In conclusion, this study proposes a robust and integrated computational workflow combining descriptor generation, QSAR modeling, applicability domain analysis, molecular docking, ADMET prediction, and molecular dynamics simulations for the rational design of potential α-glucosidase inhibitors. The findings highlight the therapeutic potential of the designed derivatives and provide a valuable in silico framework for the future development of antidiabetic agents.

## 1. Introduction

Diabetes mellitus has become a major health issue worldwide, contributing to a significant burden of disease and mortality [1]. DM is a metabolic disorder in which the body fails to maintain normal blood glucose levels, leading to hyperglycemia. The pathogenesis of diabetes involves various factors, including physical inactivity, an unhealthy diet, and genetic predisposition [2]. The main symptoms of diabetes include polyuria, polydipsia, polyphagia, weight loss, fatigue, delayed healing of wounds, blurred vision, and recurring infections [3]. Considering the prevalence of diabetes, it is imperative to look for effective preventive and control measures. The International Diabetes Federation estimates that currently there are around 532 million people living with diabetes worldwide. Moreover, this figure may rise to 783 million by the year 2045 [4]. On the other hand, the World Health Organization states that the number of people with diabetes worldwide was around 415 million in 2015. This figure may rise to 642 million by the year 2040. This indicates the rapid rise in diabetes in the world [5].

Besides the high prevalence of diabetes, the disease has the potential for causing severe complications, which affect the quality of life of the patients. These complications include heart disease, stroke, hypertension, neuropathy, nephropathy, diabetic foot, dermatological problems, blindness, gastrointestinal upsets, respiratory problems, obesity, and impaired healing of wounds, among others [6]. Two main therapeutic approaches have been identified for the management of diabetes: the inhibition of the enzymes α-amylase and α-glucosidase [7]. These two enzymes catalyze the hydrolysis of polysaccharides into monosaccharides, such as glucose, for absorption by the body. By inhibiting these two enzymes, the amount of glucose absorbed by the body from the diet decreases, reducing the blood glucose levels [8]. These two enzymes are inhibited by the commonly used antidiabetic drugs acarbose, miglitol, and voglibose [9,10,11].

Despite the therapeutic benefits of these drugs, the use of these drugs has also been associated with a number of side effects, including bloating, diarrhea, and abdominal discomfort, which has necessitated the search for new drugs for the management of diabetes [12]. Drug development is a complicated, time- and cost-intensive process, which has necessitated the use of computer-assisted drug design methodologies for the development of new drugs. These methodologies have the ability to predict the biological activity of a compound prior to its actual synthesis, making the process of developing new drugs faster [13].

Quantitative Structure–Activity Relationship (QSAR) analysis is considered one of the widely practiced computational techniques currently employed in modern drug design. The main advantage associated with the application of the QSAR approach lies in its ability to predict the biological activity of compounds directly from the structure of those compounds [14]. By establishing a mathematical correlation between the structure of the compounds and the observed activity level, the QSAR approach enables the researcher to predict the potential activity level of the newly synthesized compounds. As a result, QSAR has become a valuable approach for identifying promising drug candidates and optimizing their properties in a more efficient and cost-effective manner [15].

Several studies have investigated different classes of heterocyclic compounds such as benzimidazoles, triazoles, and thiazoles and demonstrated their strong inhibitory activity against α-amylase and α-glucosidase [16]. In 2021, Peytam et al. [17] reported the synthesis of 32 derivatives of 3-amino-2,4-diarylbenzo[4,5]imidazo[1,2-α]pyrimidine, which exhibited α-glucosidase inhibitory activity with IC_50_ values ranging from 16.4 to 297.0 μM. Their work also included molecular docking studies to better understand how these compounds interact with the target enzyme. Building on these findings, the present study uses the previously synthesized compounds and their reported IC_50_ values to develop a QSAR model aimed at identifying the key structural features responsible for α-glucosidase inhibition. Table 1 shows the two-dimensional structures of the investigated derivatives along with their corresponding IC_50_ values.

## 2. Results and Discussion

### 2.1. QSAR Study

A QSAR study was conducted using a dataset consisting of calculated descriptors and experimental pIC_50_ values for 32 compounds, aiming to develop a molecular model that predicts pIC_50_ values against alpha-glucosidase for new compounds. Initially, the ACP method was employed to reduce the number of descriptors from 303 to 190 based on matrix correlation. Those descriptors with a correlation coefficient of at least 0.95 were minimized by retaining only one of each set of correlated descriptors.

Several descriptors were identified as having a significant correlation with pIC_50_, and these were symmetric atom descriptors, energy of LUMO_+1_, shape coefficient, ATSC5e, and relative PSA. These descriptors were found to play a significant role in determining biological activity against alpha-glucosidase and should be considered when developing a model (Table S1).

### 2.2. Model Development and Validation

The dataset was divided into two sets: a test set consisting of Compounds **3i**, **3j**, **3k**, **3ab**, **3ae**, and **3ag**, and a training set consisting of the remaining compounds. The training set was used for developing and improving the QSAR model, and the test set was used as a tool for validating the model and assessing its predictive potential. The Best Model approach was used for developing the QSAR model, and multiple iterations were carried out to obtain dependable results. For assessing the quality of the QSAR model, statistical parameters such as R^2^, R^2^adj, R^2^ext, Q^2^, and RMSE were calculated. Of all the developed models, one of them possesses outstanding statistical parameters, as shown in Table 2.

The model was subjected to internal and external validation, and statistical parameters showed R^2^ = 0.834, R^2^adj = 0.803, and R^2^ext = 0.782, all greater than 0.6, while Q^2^ = 0.758, greater than 0.5, and RMSE = 0.1419. These parameters confirmed the dependability and strength of the model, as required by OECD criteria. The Variance Inflation Factor (VIF) calculation was also carried out for the model descriptors, and since the VIF is below 5, there is no significant collinearity among the descriptors.

The model was further validated using a Y-randomization (Y-scrambling) test to assess its robustness and exclude the possibility of chance correlation. The results showed markedly poor performance for the randomized models, with average r = 0.370, r^2^ = 0.150, and Q^2^ = −0.315. In contrast, the cRp^2^ value (0.763) was well above the recommended threshold, confirming the robustness and statistical reliability of the developed QSAR model (Table S2).

**Figure 1:**
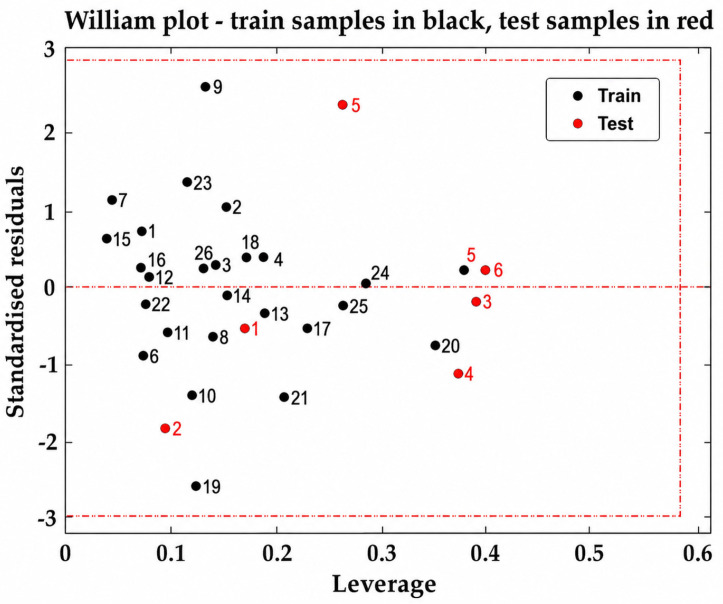
Williams plot showing the applicability domain of the model with a leverage threshold of 0.577 and standardized residual limits of ±3. Training compounds are in black and test compounds in red; the red dashed box defines the applicability domain, corresponding to the critical limits of standardized residuals (±3) and leverage (h*).

After validation, the domain of applicability was determined to specify the scope of the model in terms of the chemical space. The threshold for leverage was set at h* = 0.577 and residuals were confined to ±3. All compounds in the training and test sets were found to lie within the defined applicability domain (Figure 1), confirming that the model operates within a consistent and well-defined chemical space. This indicates that predictions generated by the model are reliable for compounds structurally related to the training set, while compounds outside this domain are not considered valid within the current QSAR model due to insufficient model support in that chemical space.

### 2.3. New Designed Compounds

**Figure 2:**
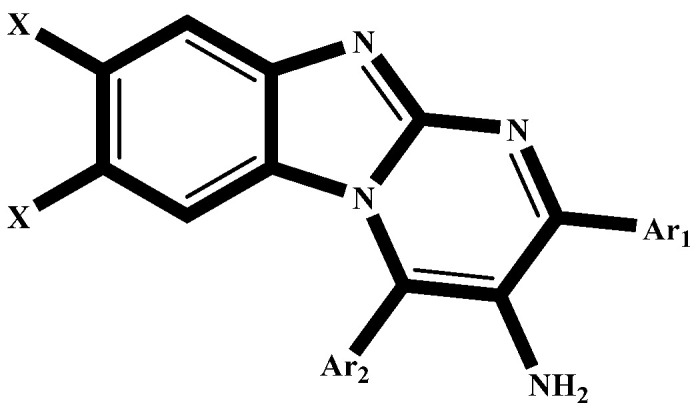
General structure of 3-amino-2,4-diarylbenzo[4,5]imidazo[1,2-α]pyrimidine derivatives, where Ar_1_ and Ar_2_ represent aryl substituents and X denotes variable substituents on the benzene ring.

Peytam et al.’s findings showed that the compounds exhibited inhibitor activity ranging from 16.4 to 297.0 μM. Compound 3k showed the highest activity against alpha-amylase, while compound 3e had the lowest. We identified that Compounds **3d**, **3h**, **3i**, **3k**, and **3ad** exhibited strong IC_50_ values. A common feature of these compounds is the presence of a chlorine (Cl) atom in the para position of the benzyl group in Ar2 (Figure 2). In contrast, the incorporation of chlorine (Cl) in position X resulted in a decrease in activity, as observed in Compounds **3aa**, **3ab**, **3ac**, **3ae**, **3af**, and **3ag**. This finding could be used as a basis for designing new derivatives.

The developed model indicates that the descriptors Next Lowest Unoccupied Molecular Orbital (LUMO_+1_), symmetric atoms, Autocorrelation of Topological Structure (Lag 5), Weighted by Electronegativity (ATSC5e), Geary Autocorrelation of (Lag 3), Weighted by Polarizability (GATS3p) have the greatest influence on the desired biological activity. Changing these descriptors can be an effective way to influence the pIC_50_ value. The *t*-test results for these descriptors are −3.9997, 8.574, −5.373, and 5.161, respectively.

The descriptor LUMO_+1_, which represents the molecule’s ability to accept electrons, shows that as the value for this descriptor decreases, the molecule becomes more inclined to accept electrons, thus increasing the reactivity of the molecule. The negative value for the *t*-test indicates that as the descriptor for LUMO_+1_ decrease; the pIC_50_ value will increase, thus increasing the reactivity of the compound. The descriptor for symmetric atoms represents the symmetrical arrangements of atoms within the molecule. An increase in the pIC_50_ value indicates that the more the symmetric the atoms, the higher the reactivity of the compound.

The descriptor ATSC5e represents the overall influence of the electronic effect within the molecule. A negative value for the *t*-test indicates that as the descriptor for ATSC5e decreases, the pIC_50_ value will increase, thus increasing the reactivity of the compound. Finally, GATS3p, which measures the distribution of atomic properties weighted by polarizability, indicates that increasing this descriptor enhances activity, as a positive *t*-test value correlates with improved inhibitory properties. These findings underscore the significance of modifying key descriptors to enhance the activity of the compounds.

Based on this analysis, we proposed seven new promising compounds, and the developed model was utilized to predict their pIC_50_ values. The proposed compounds, along with their respective model descriptors and predicted pIC_50_ values, are shown in Table 3. In order to verify the accuracy of the predictions, the leverage of each compound was calculated to check whether it falls within the applicability domain of the model.

### 2.4. Molecular Docking

To reveal the binding modes and possible inhibitory potential of the newly designed compounds, molecular docking simulations were carried out, and the results are presented in Table 4 and Table 5. The co-crystal structure of alpha-glucosidase (PDB code: 2F6D), complexed with the known inhibitor and reference drug in the treatment of diabetes, acarbose, has been used to reveal the binding site, key residues at the active site, and the nature of the interactions involved in the enzymatic inhibition process. As described in the literature [18], the catalytic residues forming the active site in this complex are Asp70, Arg69, Arg345, Glu210, Glu211, and Glu456.

**Figure 3:**
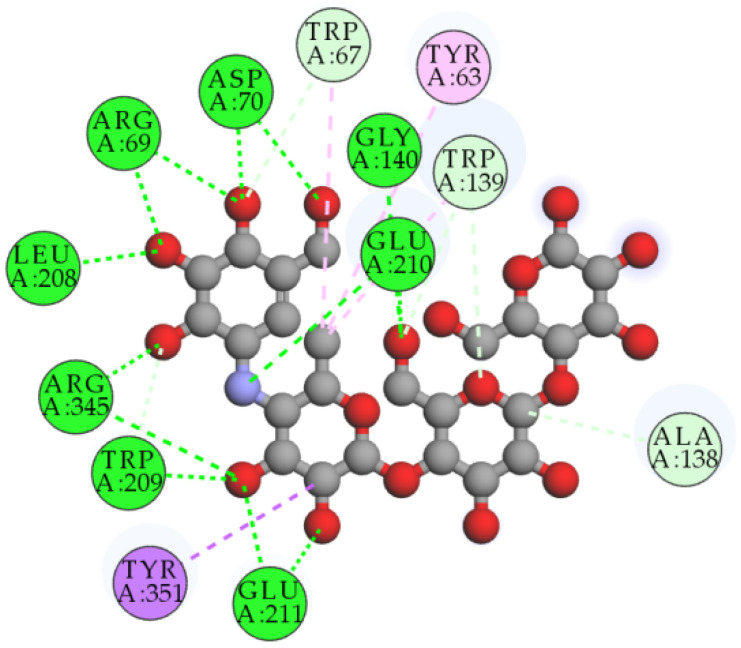
Main interacting residues of the 2F6D receptor in complex with Acarbose Green, light green, pink, and purple lines represent conventional hydrogen bonds, carbon–hydrogen bonds, alkyl interactions, and π–sigma interactions, respectively.

The analysis of the co-crystallized complex corroborated these findings and showed that acarbose forms multiple hydrogen bonds with Asp70, Arg69, Arg345, Glu210, Glu211, Leu208, Trp209, and Glu456, as well as a π-sigma interaction with the aromatic residue Tyr351 (Figure 3). These findings suggest that these residues are critical for alpha-glucosidase inhibition and act as key targets and references for evaluation of interactions formed with other designed molecules.

**Figure 4:**
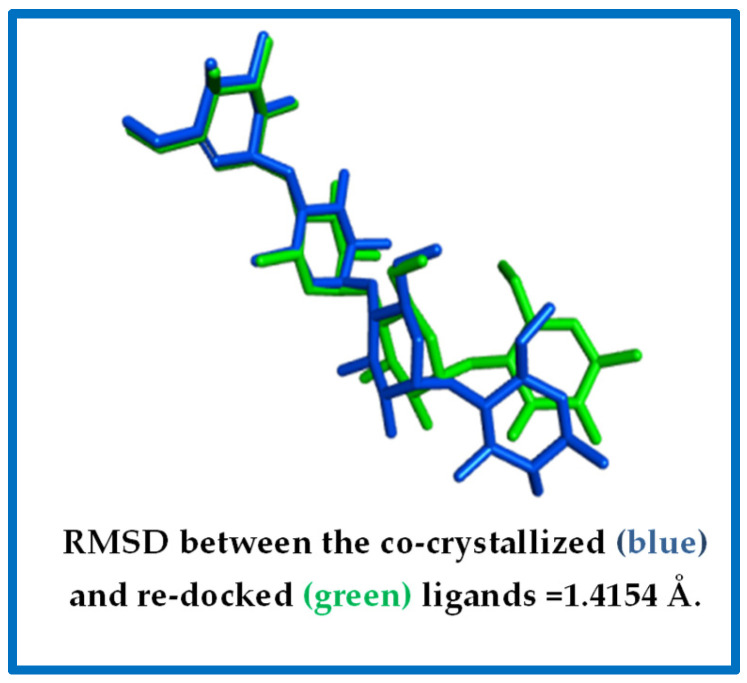
Superposition of the co-crystallized (blue) and re-docked (green) ligands, confirming the validity of the docking protocol with an RMSD value of 1.4154 Å.

In order to validate the accuracy of the docking protocol, a superposition was performed between the conformation of acarbose in the co-crystallized complex (PDB ID: 2F6D) and the pose obtained after re-docking. This comparison allowed for the evaluation of the algorithm’s ability to accurately reproduce the ligand’s orientation and interactions within the active site. The two conformations were found to be highly similar, with a satisfactory overlap and conservation of the key interactions previously identified. This good alignment, quantified by a low RMSD value of 1.4154 Å (<2) (Figure 4), confirms the reliability of the docking protocol for predicting the binding modes of newly designed molecules within the alpha-glucosidase active site.

Molecular docking of these newly designed compounds with alpha-glucosidase showed binding affinities of these compounds, ranging from −9.1 kcal/mol (**ND28**) to −10.9 kcal/mol (**ND8**), whereas for the standard inhibitor, it was −9.2 kcal/mol for acarbose. These results suggest that these designed compounds have strong binding affinities with the active site of alpha-glucosidase and thus have potential inhibitory effects similar to or greater than those of acarbose.

The binding mode of acarbose observed in the present docking study closely matches the interaction pattern reported in the literature for the co-crystallized complex, indicating a consistent and reliable reproduction of the native binding pose. As shown in Figure 3, the docked acarbose adopts a highly similar orientation within the α-glucosidase active site and preserves the key interaction network. It forms multiple hydrogen bonds with essential residues, including Asp70, Arg69, Arg345, Glu210, Glu211, Leu208, Trp209, and Glu456, in addition to a π–sigma interaction with Tyr351. The strong agreement between the docked pose and the reported binding mode confirms the validity of the docking protocol and supports the relevance of these residues as critical determinants for ligand recognition and inhibitory activity against α-glucosidase.

Likewise, the designed compounds have been found to have a range of significant interactions with the catalytic residues, including conventional hydrogen bonds with Glu210 (2.02 Å) and Trp209 (2.85 Å) for **ND9**; π-alkyl interactions with Trp362 and Trp473; π-anion interactions with Glu210 (3.52 Å) and Glu456 (3.87 Å); and π-π stacking and T-shaped interactions with Trp139 and Tyr63. **ND8** engaged in a hydrogen bond with Trp209 (2.48 Å), π-anion interactions with Glu210 (3.26 Å), Glu211 (3.65 Å), and Glu456 (4.18 Å), and multiple hydrophobic and aromatic contacts including π-alkyl interactions with Trp209, Trp362, Tyr351, and Tyr63, and π-π interactions involving Tyr351 and Trp67. **ND14** formed hydrogen bonds with Asp70 (2.95 Å), Glu210 (3.09 Å), and Trp209 (2.40 Å), π-anion interactions with Glu210, Glu211, and Glu456, and π-donor and aromatic stacking interactions with Trp67, Tyr351, Tyr63, and Trp139. **ND24** displayed a hydrogen bond with Trp209 (2.50 Å), π-anion interactions with Glu210 (3.28 Å), Glu211 (3.65 Å), and Glu456 (4.14 Å), and extensive π-alkyl and aromatic interactions involving Trp209, Trp362, Tyr351, Tyr63, and Trp67.

**ND28** established hydrogen bonds with Arg345 (2.59 Å), Glu211 (2.69–2.83 Å), Tyr351 (2.96 Å), and Tyr63 (2.55 Å), π-anion interactions with Glu210 and Glu211, and π-π stacking and T-shaped interactions with Tyr351, Trp139, and Trp362. **ND33** formed hydrogen bonds with Tyr351 (2.95 Å) and Trp209 (2.61 Å), π-anion interactions with Glu210, Glu211, and Glu456, π-alkyl interaction with Trp473, and aromatic stacking with Tyr351 and Tyr63. **ND37** engaged in hydrogen bonds with Asp70 (2.42 Å), Glu210 (2.63–2.90 Å), Glu211 (2.96 Å), and Trp209 (2.45 Å), π-anion interactions with Glu210, Glu211, and Glu456, π-donor hydrogen bonding with Trp67, and π-π stacking and T-shaped interactions with Tyr351, Tyr63, and Trp139.

In all the designed molecules, multiples interactions have been observed, which are common with acarbose. These include conventional hydrogen bonds and π-interactions with the essential catalytic residues, including Trp209, Glu210, Glu211, Asp70, Tyr351, Trp139, and Trp67.

In conclusion, based on the molecular docking results, it can be concluded that the newly designed molecules have high binding affinity and are able to establish essential interactions within the active site of alpha-glucosidase. It has been observed that these molecules have the potential to act as efficient enzyme inhibitors which are at least, if not more, effective than acarbose. Thus, the potential of these molecules in treating diabetes can be regarded as high.

### 2.5. ADMET Analyses

To determine the best compounds, a detailed evaluation of their physicochemical properties was conducted. This evaluation was done based on Lipinski’s rule of five, a set of criteria for predicting oral bioavailability of drug compounds [3]. Based on the evaluation, it is evident from Table 6 that the majority of the compounds follow Lipinski’s criteria, indicating good potential for oral absorption. However, for molecule 24, it is evident that it fails two of the four criteria, having a molecular weight of 504.2 g·mol^−1^ and a log P of 6.57, which are above the recommended limits.

With regard to the compounds that fulfilled the requirements for oral bioavailability, pharmacokinetic parameters were predicted as shown in Table 7, and this began with absorption. For compound **9** and **ND33**, it was observed that the intestinal absorption rate was 100%, which is a very good sign of oral bioavailability. This was followed by **ND8, ND28, ND14**, and **ND37**, with absorption values of 89.8%, 89.78%, 83.92%, and 74.74%, respectively. The Caco-2 cell membrane permeability values are indicative of penetration through the intestinal barrier, and values ranged from −0.02 for **ND9** up to −0.79 for **ND14**. Finally, with regard to skin permeability, it was observed that all compounds had similar values of around −2.735 cm/h.

In terms of distribution, compound **ND8** showed the highest permeability values across the BBB and into the CNS, with scores of 0.48 and −1.20, respectively, suggesting potential brain penetration. Conversely, **ND28** had the lowest BBB permeability (−1.97), while **ND14** and **ND28** also showed the lowest CNS permeability values of −3.77 and −3.60, respectively, indicating limited distribution to the brain.

With respect to metabolism, only compounds **ND9, ND8**, and **ND33** were predicted to be substrates of the CYP3A4 enzyme, suggesting these molecules may be metabolized by this major cytochrome P450 isoform. Additionally, inhibition of CYP1A2, CYP2C19, and CYP2C9 was predicted for compounds **ND8, ND9, ND24, ND33**, and **ND37**. Notably, all the investigated compounds are predicted to inhibit CYP3A4, which is a key isoenzyme of the cytochrome P450 system involved in the metabolism of a large proportion of clinically used drugs [19,20]. This prediction suggests a potential risk of drug–drug interactions, as CYP3A4 inhibition may reduce the metabolic clearance of co-administered substrates, leading to increased systemic exposure, prolonged half-life, and a possible risk of toxicity [21,22].

Regarding excretion, the total clearance was predicted to range from 0.49 (**ND9**) to 0.82 (**ND37**), indicating a moderate elimination rate for the tested compounds.

Finally, to assess the safety profile of these ligands, toxicity predictions were conducted. All compounds were found to be non-hepatotoxic and non-skin-sensitizing, except for **ND28** and **ND37**, which showed potential hepatotoxicity, suggesting a need for further toxicological evaluation in future development stages.

Although the ADMET predictions obtained in this study provided valuable insight into the pharmacokinetic and toxicity profiles of the investigated compounds, these computational estimations remain dependent on the algorithms and datasets implemented in the prediction platforms [23]. Therefore, the application of multiple ADMET prediction tools and consensus-based cross-validation may further improve the robustness and reliability of the obtained results [24,25]. In addition, while most compounds satisfied Lipinski’s rule of five, some compounds exhibited minor rule deviations that could influence their oral bioavailability and drug-likeness properties. Nevertheless, such violations do not necessarily preclude biological activity, as several clinically approved drugs are known to violate one or more Lipinski criteria while still exhibiting favorable pharmacological performance [26].

### 2.6. Molecular Dynamics

Following a virtual screening study aimed at identifying lead compounds with promising activity and favorable pharmacokinetic profiles, three candidates were selected: **ND8, ND33**, and **ND37**. To determine the stability of these molecules under a biological system mimic, a molecular dynamics simulation was employed for 100 nanoseconds. Molecular dynamics can be used for the prediction of ligand and protein dynamics during the course of the simulation by calculating various parameters [16].

For instance, the root mean square deviation (RMSD) can be used to obtain information regarding the stability of the protein–ligand complex, while the root mean square fluctuation (RMSF) can be used to obtain information regarding the flexibility of the amino acids within the protein. The radius of gyration (rGyr) can also be used to obtain information regarding the dynamics of the protein structure, and the solvent-accessible surface area (SASA) can be used to obtain information regarding the protein surface and the dynamics of the ligand–protein interaction [27].

### 2.7. Protein RMSD Analysis

**Figure 5:**
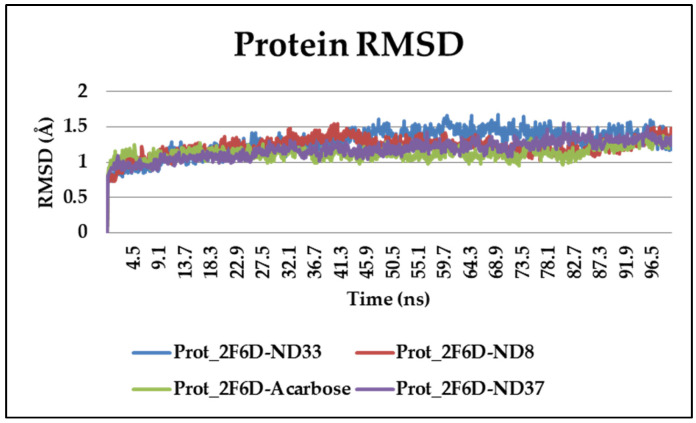
Time-dependent RMSD of 2F6D protein Cα atoms in complex with ND8, ND33, ND37 and acarbose.

As shown in Figure 5, all protein–ligand complexes, including the reference compound acarbose, exhibited structural stability throughout the 100 ns molecular dynamics simulation. The RMSD profiles remained below 1.8 Å, indicating that no major conformational changes occurred during the simulation. Overall, all complexes displayed comparable stability, with RMSD values fluctuating within a narrow range of approximately 1.0–1.7 Å, suggesting stable binding interactions and well-equilibrated protein–ligand systems. These results demonstrate that the investigated ligands remained firmly accommodated within the binding pocket throughout the simulation period, maintaining the structural integrity of the complexes.

### 2.8. Ligand RMSD Analysis

**Figure 6:**
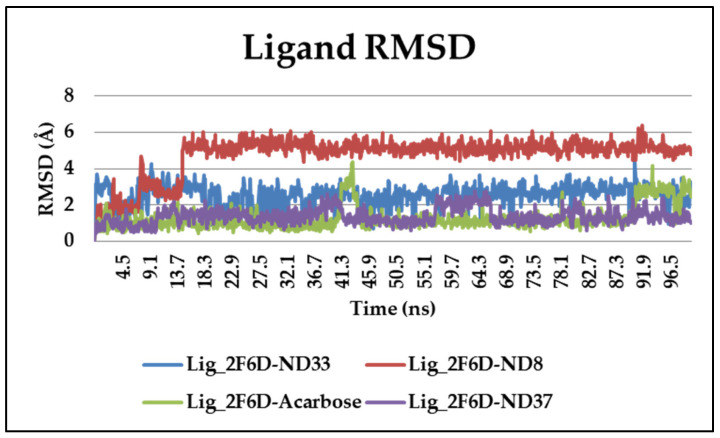
Time-dependent ligand RMSD of the complexes studied: **ND8**-2F6D, **ND33**-2F6D, **ND37**-2F6D and acarbose-2F6D.

The root mean square deviation of the ligands within the 2F6D binding pocket is presented in Figure 6. Overall, most protein–ligand complexes exhibited stable behavior throughout the 100 ns simulation, with ligand RMSD values generally ranging between 2 and 4 Å and showing only minor fluctuations, indicating that the ligands remained well accommodated within the active site. Among the investigated compounds, **ND33, ND37**, and acarbose displayed particularly stable trajectories, maintaining consistent RMSD profiles within this range and suggesting stable binding interactions throughout the simulation period. In contrast, **ND8** exhibited comparatively higher mobility within the binding pocket. During the first 15 ns of the simulation, its RMSD values fluctuated between approximately 3 and 4 Å, followed by an increase and stabilization between 5 and 6 Å. Despite this increased flexibility, **ND8** remained associated with the binding pocket throughout the simulation, suggesting the maintenance of ligand–protein interactions after structural readjustment.

### 2.9. Protein RMSF Analysis

**Figure 7:**
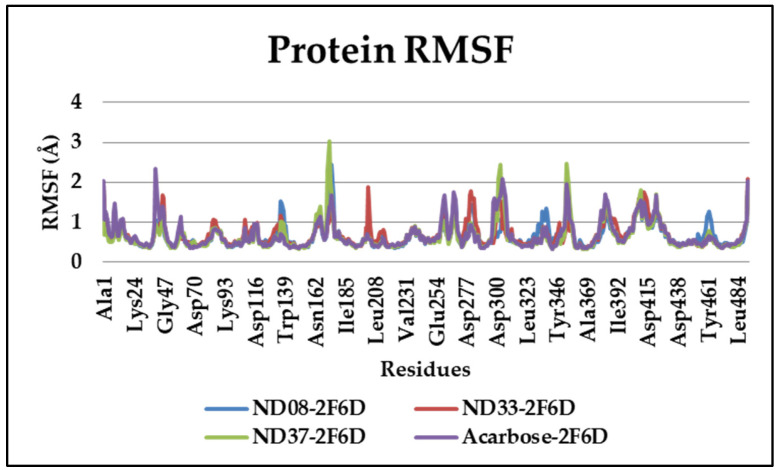
The protein RMSF of 2F6D protein Cα atoms in complex with ND8, ND33, ND37 and acarbose.

The root mean square fluctuation analysis revealed that all protein–ligand complexes exhibited stable behavior throughout the simulation, with residue fluctuations remaining below 3.0 Å (Figure 7). This indicates that ligand binding did not induce significant structural flexibility in the protein. Overall, the RMSF profiles demonstrated limited residue mobility and suggest that all complexes maintained their structural integrity and stability during the simulation period.

### 2.10. Ligand–Protein Interaction Analysis

**Figure 8:**
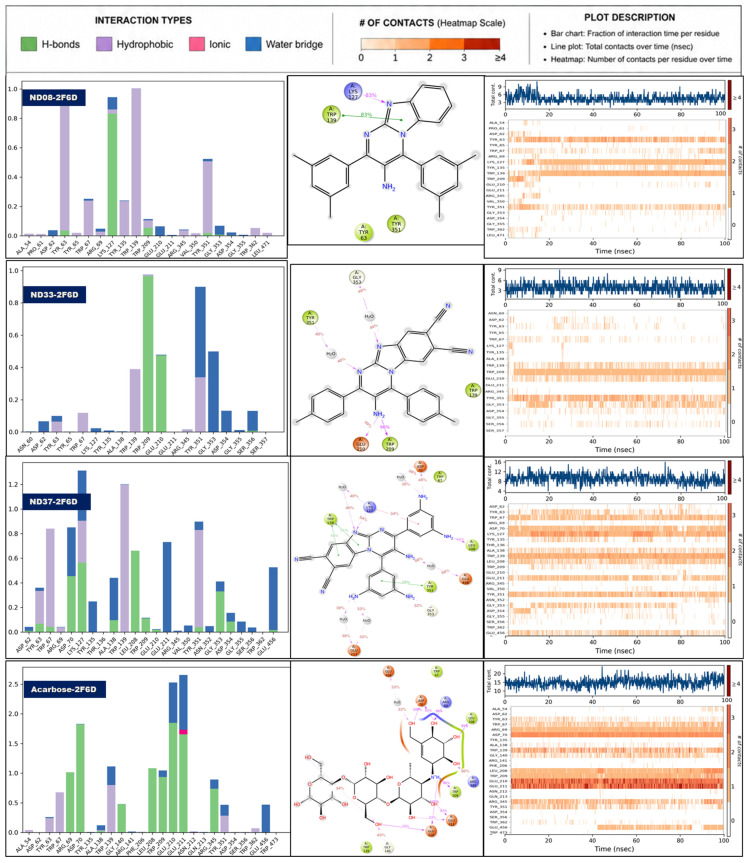
Protein–ligand interaction fraction plots, timeline and 2D visualization of ND08, ND33, ND37 and acarbose complexes during molecular dynamics simulation.

The protein–ligand interaction analysis derived from the interaction histograms, timelines, and 2D interaction maps revealed the most persistent contacts formed during the molecular dynamics simulations. As presented in Figure 8, acarbose established an extensive network of hydrogen bonds with Arg69, Asp70, Leu208, Trp209, Glu210, and Glu211, together with hydrophobic interactions involving Trp67 and Trp139 and water-mediated bridges with Glu210 and Glu211. **ND08** was primarily stabilized through hydrophobic contacts with Tyr63, Trp139, and Tyr351, along with a hydrogen bond with Lys127. **ND33** maintained hydrophobic interactions with Trp139 and Tyr351, hydrogen bonds with Trp209 and Glu210, and water bridges involving Tyr351 and Gly353. Among the designed compounds, **ND37** exhibited the most extensive interaction network, including hydrophobic contacts with Trp67, Trp139, and Tyr351, hydrogen bonds with Asp70, Lys127, Leu208, and Gly353, and water-mediated interactions with Glu211 and Glu456. These persistent interactions, illustrated in Figure 8, contributed significantly to the stability of the respective protein–ligand complexes throughout the simulation period.

### 2.11. RGyr and SASA Analysis

**Figure 9:**
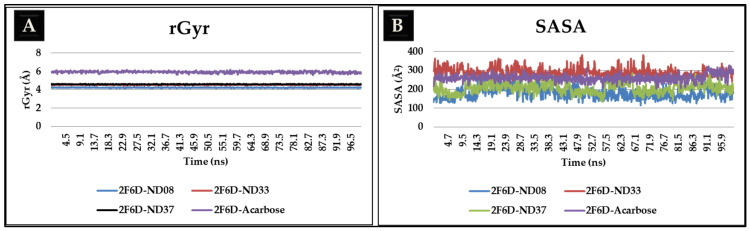
Time-dependent rGyr (**A**) and SASA (**B**) of the studied complexes: ND8-2F6D, ND33-2F6D, ND37-2F6D and acarbose-2F6D.

The radius of gyration and solvent-accessible surface area analyses were performed to evaluate the compactness and solvent exposure of the protein–ligand complexes during the molecular dynamics simulations (Figure 9). The rGyr results demonstrated that the **ND08**–2F6D complex exhibited the lowest average value (4.229 Å), indicating the highest degree of structural compactness among the investigated systems. The **ND33**–2F6D and **ND37**–2F6D complexes displayed comparable average rGyr values of 4.541 Å and 4.593 Å, respectively, suggesting stable conformational behavior throughout the simulation. In contrast, the acarbose–2F6D complex showed the highest average rGyr value (5.930 Å), reflecting a comparatively less compact structure.

The SASA analysis revealed notable differences in the extent of solvent exposure among the complexes. The **ND08**–2F6D complex exhibited the lowest average SASA value (179.333 Å^2^), followed by **ND37**–2F6D (206.535 Å^2^), indicating reduced solvent accessibility and favorable accommodation within the binding cavity. The acarbose–2F6D complex displayed an intermediate SASA value of 261.514 Å^2^, whereas **ND33**–2F6D showed the highest average SASA value (288.624 Å^2^), suggesting greater exposure to the solvent environment.

Collectively, the rGyr and SASA results indicate that **ND08** forms the most compact and least solvent-exposed complex with 2F6D, while **ND37** also exhibits favorable burial within the binding pocket. In comparison, the reference inhibitor acarbose displayed a less compact structure and higher solvent accessibility than **ND08** and **ND37**. These findings support the potential of the designed compounds, particularly **ND08** and **ND37**, to establish stable interactions within the active site of the target protein.

### 2.12. MM/GBSA Binding Free Energy

The MM/GBSA binding free energy results (Table 8) were calculated for the selected protein–ligand complexes involving acarbose, **ND8, ND33**, and **ND37** bound to the 2F6D receptor. The obtained negative ΔG values indicate favorable binding interactions for all evaluated complexes, where more negative values correspond to stronger binding affinity and enhanced complex stability.

The MM/GBSA binding free energy calculations further supported the stability of the protein–ligand complexes. As shown in Table 8, all investigated compounds exhibited negative binding free energy values, indicating thermodynamically favorable interactions with the 2F6D target. The reference ligand acarbose displayed the most favorable binding free energy (ΔG = −74.35 kcal/mol), followed by **ND08** (ΔG = −58.06 kcal/mol), **ND37** (ΔG = −51.49 kcal/mol), and **ND33** (ΔG = −41.41 kcal/mol). Although the designed compounds exhibited lower binding affinities than the reference ligand, their negative ΔG values indicate stable binding within the active site. Among the designed compounds, **ND08** and **ND37** demonstrated the strongest binding affinity, suggesting its potential as a promising inhibitor of the 2F6D target.

Despite the promising results obtained in this study, certain limitations should be acknowledged. The QSAR model was developed using a relatively small dataset, which may limit the generalizability and predictive performance of the model for structurally diverse compounds outside the investigated chemical space [28]. In addition, the present work is entirely based on computational approaches, including QSAR modeling, molecular docking, ADMET prediction, MM/GBSA calculations, and molecular dynamics simulations, without experimental validation. Therefore, the predicted biological activities and pharmacokinetic properties of the designed compounds require confirmation through in vitro and in vivo studies [29]. Although the designed compounds demonstrate promising α-glucosidase inhibitory potential along with favorable docking and molecular dynamics stability, their predicted CYP3A4 inhibitory profile represents a limitation in terms of pharmacokinetic safety and drug-likeness. Therefore, further structural optimization and experimental validation are required to mitigate CYP3A4 inhibition while preserving biological activity. Furthermore, the molecular docking and molecular dynamics analyses were performed using a single receptor structure, which may not fully represent the conformational flexibility of the target enzyme under physiological conditions [30]. Consequently, additional experimental and computational investigations are necessary to further validate and optimize the proposed compounds as potential α-glucosidase inhibitors.

## 3. Material and Methods

### 3.1. Quantitative Structure–Activity Relationship

The 32 synthesized compounds from the previous study by Peytam et al. in 2021 [17] were employed in the present study to apply the QSAR method [31]. These compounds were drawn, optimized under MMFF94 force field and then saved in SDF format. Several programs, including Chem3D 19.0, Data Warrior 05.05.00 [32], Gaussian 09W [33], and PaDEL 2.18 [34], were used to calculate a total of 303 molecular descriptors. These descriptors provide a structural characterization of each molecule. The IC_50_ value indicates the concentration of a compound needed to inhibit 50% of alpha-glucosidase activity. To facilitate easier comparison and interpretation, the IC_50_ values were converted into pIC_50_ values using the formula -log (IC_50_), which helps standardize the data.

### 3.2. Principal Component Analysis 

The dataset, which included each molecule with its calculated descriptors and pIC_50_ values, was analyzed using principal component analysis (PCA) to reduce the dataset size. PCA was applied to eliminate redundant, correlated descriptors while retaining essential information, allowing for the identification of key descriptors influencing the studied activity [35,36]. The correlation matrix was used to assess the relationships between descriptors. This analysis was performed using the XLSTAT 2014.5.03 program [36].

### 3.3. Multiple Linear Regression

The relationship between calculated molecular descriptors and alpha-glucosidase inhibitor activity was modeled using multiple linear regression (MLR) [14]. This is a statistical technique used to quantify the relationship between the calculated descriptors and biological activity. MLR is used to predict biological activity from the molecular structure of a compound. In this technique, the relationship is used to understand which descriptors have a significant effect on biological activity [37]. This model can be used to predict new compounds with desired properties. Data were split into a training set comprising 80% of the data (n = 26) and a test set comprising 20% (n = 6), corresponding to a 4:1 (or 1/5 for test) ratio. The number of descriptors used varied from 3 to 5. The best model was obtained using a tolerance of 0.0001 and a confidence interval of 95%. Model evaluation parameters included fitting parameters, internal and external validation, Variance Inflation Factor, and randomization. As per OECD guidelines [38], parameters such as R^2^, R^2^_adj_, R^2^_ext_, Q^2^, VIF, and OECD compliance must meet a set of threshold values, as shown in Table 9 [39,40].

The domain of applicability of the chosen model was evaluated using Williams’ plot, a graphical tool used to assess whether the compound in question falls within the domain where the model is considered to be valid and accurate. Williams’ plot also helps in the identification of compounds that are considered to be outside the domain of applicability and the prediction capability of the model [41].

For the generation of Williams’ plot, Matlab R2023a was used to calculate the leverage values and the standardized residuals for each compound in the data set [42]. Compounds that have high leverage values and high residuals are considered to be outside the domain of applicability and are therefore considered to be outside the model. This ensures that the model is accurate and that predictions are made for compounds that fall within the domain of applicability [43].

### 3.4. Molecular Docking Study

A molecular docking study was carried out using Autodock Vina software (Version 1.5.6) [44], where the proposed compounds were evaluated for their interaction with the active site of alpha-glucosidase enzyme and analyzed for their potential to form complexes with the receptor. This method generates multiple poses of the ligand, along with binding scores that reflect the quality of the formed complex.

**Figure 10:**
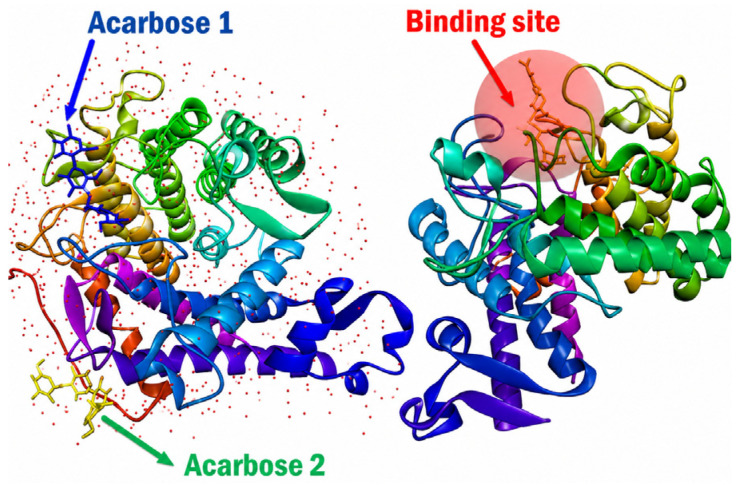
3D structure of the alpha-glucosidase receptor complexed with acarbose, highlighting the defined binding site.

The target receptor was retrieved from the Protein Data Bank (PDB) with the PDB ID: 2F6D. The selection of this receptor was based on its high resolution and its co-crystallization with the reference drug, acarbose, which facilitated the identification of the binding site and key residues [18]. By using Autodock tools [45], the protein preparation was carried out as described in our previous studies by adding missing atoms, performing energy minimization, removing water molecules and heteroatoms, adding polar hydrogens, and assigning charges. The binding site was determined based on the initial position of the co-crystallized ligand, acarbose. A cubic box with dimensions of X = Y = Z = 20 Å and a center at coordinates X = 12.68 Å, Y = 10.80 Å and Z = −6.35 Å was used to cover the receptor’s binding site as seen in Figure 10.

The proposed compounds were drawn and saved in PDB format after optimization using Avogadro software (Version 1.2.0) [46]. Prior to docking, a re-docking of the co-crystallized ligand was performed to validate the docking protocol. An RMSD value of less than 2 Å between the co-crystallized and re-docked ligands confirms the reliability of the docking procedure. Docking results were interpreted using Discovery Studio Visualizer (Version 20.1.0.19295) [47], which provided a 2D representation of the resulting ligand–receptor complex.

### 3.5. ADMET Analysis

Poor pharmacokinetic and pharmacodynamic properties are often the primary reasons for the withdrawal of potential medications at the preclinical stage. These properties predict the behavior of the studied compounds in the human body through parameters such as absorption, distribution, metabolism, excretion, and toxicity. Additionally, several rules, including Lipinski, Veber, Egan, Ghose, and Muegge, define threshold values to determine whether a compound has good oral bioavailability [48].

In this study, an ADMET analysis was conducted for the proposed compounds to assess their oral bioavailability. The pKCSM online tool (https://biosig.lab.uq.edu.au/pkcsm/, accessed 5 June 2026) was used to calculate key pharmacokinetic parameters such as intestinal absorption, solubility, Caco-2 permeability, volume of distribution (Vdss), central nervous system (CNS) permeability, blood–brain barrier (BBB) permeability, and Minnow toxicity. These parameters were used to evaluate the suitability of the proposed compounds for further development [49].

### 3.6. Molecular Dynamics Simulation

Molecular dynamics (MD) simulations were performed on the best-selected compounds, using the protein–ligand complexes obtained from molecular docking as the initial structures. This approach enabled the evaluation of the interaction strength between the ligands and the target protein, as well as the assessment of complex stability through several parameters, including RMSD, RMSF, and ligand property analyses.

MD simulations were carried out using Desmond (Schrödinger Release 2025-4, Schrödinger LLC, New York, NY, USA) integrated within Maestro 12.5.139. The protein–ligand systems were modeled using the OPLS force field under periodic boundary conditions within an NPT ensemble. Initially, each complex was solvated in an orthorhombic box containing TIP3P water molecules with a buffer distance of 10 Å. The systems were subsequently neutralized by adding Na^+^ and Cl^−^ ions at a concentration of 0.15 M to mimic physiological conditions. A simulation time step of 2 fs was applied throughout the simulations. Short-range non-bonded interactions, including van der Waals and electrostatic interactions, were calculated using a cutoff radius of 9.0 Å, while long-range electrostatic interactions were treated using the Particle Mesh Ewald (PME) method. The system temperature was maintained at 300 K using the Nose–Hoover thermostat, whereas pressure was regulated at 1 atm using the Martyna–Tobias–Klein barostat [50].

Following system preparation, equilibration was first performed under the NVT ensemble for 1 ns, followed by a production phase under the NPT ensemble for 100 ns. These simulation conditions ensured adequate equilibration, stability, accuracy, and reproducibility of the molecular dynamics trajectories, providing reliable data for subsequent analyses of protein–ligand interactions and complex stability [51].

## 4. Conclusions

This study aims to introduce an advanced integrated in silico approach that combines different techniques in molecular modeling and computational chemistry to identify, optimize, and validate 3-amino-2,4-diarylbenzo[4,5]imidazo[1,2-α]pyrimidine derivatives as promising alpha-glucosidase inhibitors. The application of a complete set of molecular descriptors calculated with different software packages made it possible to apply principal component analysis to simplify the complex problem and identify the most important features in it. The predictive model developed by the application of multiple linear regression was found to have strong prediction ability, and the reliability of the model was ensured through rigorous internal and external validation procedures.

The precise determination of the applicability domain of the developed model helped to assess the relevance of the newly proposed compounds, among which seven new derivatives were found to possess higher predicted values of pIC_50_ than those of the most active molecules in the studied set. These compounds are found to be located within the applicability domain, which makes them suitable candidates for the design of new therapeutic agents for the treatment of type 2 diabetes. Moreover, the results of the molecular docking study provided valuable insights into the binding behavior of the novel inhibitors with the alpha-glucosidase enzyme, which helped in understanding the mechanism of action of the new inhibitors. The pharmacokinetic potential of the new compounds, which is one of the key aspects of designing new therapeutic agents, has also been validated by the ADMET study.

The molecular dynamics simulations further improved the understanding of the stability of enzyme-inhibitor complexes over time, thus improving the understanding of the efficacy of the inhibitors in binding to the enzyme. The MM/GBSA binding free energy calculations further confirmed the strong binding affinity and thermodynamic stability of the selected compounds toward the alpha-glucosidase active site, supporting the reliability of the docking and molecular dynamics results. The application of the integrated methodology has provided a strong platform for the rapid and efficient identification of drug candidates, as seen in the development of inhibitors of alpha-glucosidase for the management of type 2 diabetes. The promising results form the basis for subsequent experimental verification, which is essential in moving forward with therapeutic applications of these predictions made by computer simulations. This study again highlights the importance of computer simulations in modern pharmaceutical research, which significantly reduces the cost and time scale involved in drug design.

## Figures and Tables

**Table 1:** 2D structures of the studied compounds along with their experimental IC_50_ values, as reported by Peytam et al. (2021) [17].

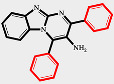 (**3a**) IC_50_ = 53.8 μM		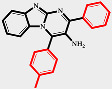 (**3b**) IC_50_ = 37.8 μM	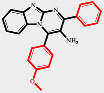 (**3c**) IC_50_ = 85.3 μM		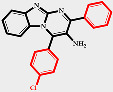 (**3d**) IC_50_ = 26.7 μM
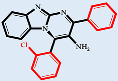 (**3e**) IC_50_ = 297.0 μM		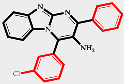 (**3f**) IC_50_ = 193.8 μM	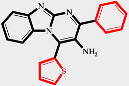 (**3g**) IC_50_ = 91.3 μM		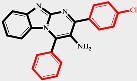 (**3h**) IC_50_ = 42.6 μM
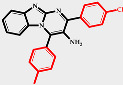 * (**3i**) IC_50_ = 36.7 μM		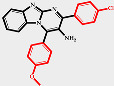 * (**3j**) IC_50_ = 114.3 μM	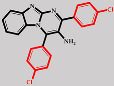 * (**3k**) IC_50_ = 16.4 μM		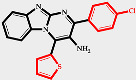 (**3l**) IC_50_ = 28.0 μM
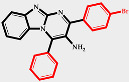 (**3m**) IC_50_ = 62.7 μM		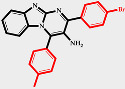 (**3n**) IC_50_ = 48.4 μM	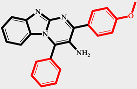 (**3o**) IC_50_ = 75.4 μM		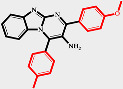 (**3p**) IC_50_ = 65.4 μM
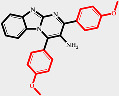 (**3q**) IC_50_ = 122.7 μM		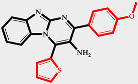 (**3r**) IC_50_ = 128.4 μM	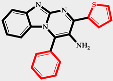 (**3s**) IC_50_ = 160.0 μM		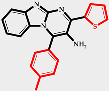 (**3t**) IC_50_ = 188.5 μM
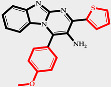 (**3u**) IC_50_ = 222.8 μM		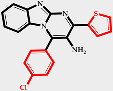 (**3w**) IC_50_ = 136.0 μM	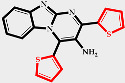 (**3x**) IC_50_ = 254.7 μM		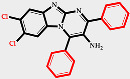 (**3y**) IC_50_ = 78.4 μM
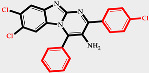 (**3z**) IC_50_ = 123.6 μM		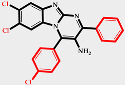 (**3aa**) IC_50_ = 64.5 μM	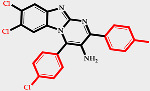 * (**3ab**) IC_50_ = 141.0 μM		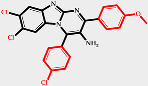 (**3ac**) IC_50_ = 224.2 μM
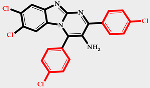 (**3ad**) IC_50_ = 48.4 μM		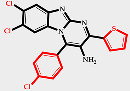 * (**3ae**) IC_50_ = 72.9 μM	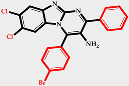 (**3af**) IC_50_ = 85.4 μM		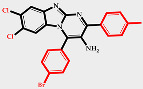 * (**3ag**) IC_50_ = 102.6 μM
	Lowest IC_50_ value	*	Test compound		Highest IC_50_ value

**Table 2:** The developed QSAR model and calculated parameters which prove the validity of the model.

Equation	pIC_50_ = −2.38908 − 0.54889 × LUMO_+1_ + 0.24822 × Symmetric atoms − 0.85240 × ATSC5e + 4.98104 × GATS3p							
R	0.913	R^2^_ext_	0.782	Descriptors	LUMO_+1_	Symmetric atoms	ATSC5e	GATS3p
R^2^	0.834	Q^2^	0.758	VIF	1.1988	1.389	1.282	1.656
R^2^_adj_	0.803	RMSE	0.1419	T_test_	−3.9997	8.574	−5.373	5.161

**Table 3:** The designed compounds, calculated molecular descriptors, predicted values of pIC_50_, and leverage values according to the developed QSAR model.

ND	Structure	LUMO^+1^	Symmetric atoms	ATSC5e	GATS3p	pIC_50_	h_i_
ND8	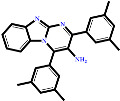	−0.6634	6	−0.0886	1.1667	5.351	0.4732
ND9	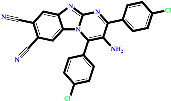	−1.7685	4	−0.2660	1.0465	5.014	0.4941
ND14	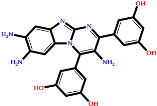	−0.6196	6	−0.2411	1.1147	5.198	0.4131
ND24	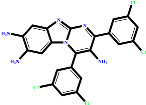	−1.4460	6	0.3238	1.0980	5.087	0.5083
ND28	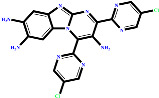	−1.6095	4	0.0608	1.1928	5.377	0.2754
ND33	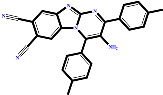	−1.5344	4	0.0031	1.0827	4.836	0.2365
ND37	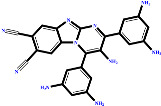	−1.3948	6	−0.0015	1.1233	5.462	0.2700

**Table 4:** The predicted binding energy values in kcal/mol and the 2D visualization of the formed complexes from the molecular docking study.

ND	Score kcal/mol	2D Visualization	ND	Score kcal/mol	2D Visualization
ND9	−9.7	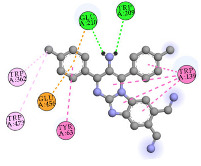	ND28	−9.1	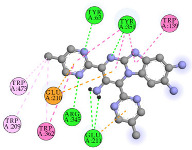
ND8	−10.9	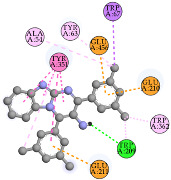	ND33	−9.8	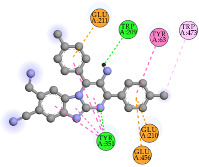
ND14	−9.4	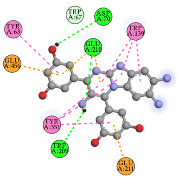	ND37	−9.8	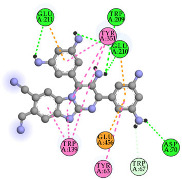
ND24	−10.1	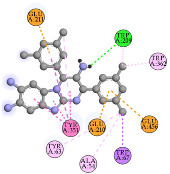	Acarbose	−9.2	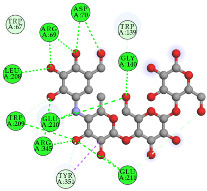
Pi-Anion Pi-Cation		Conventional H-Bond			Pi-Pi Stacking Pi-Pi T-Shaped
Pi-Sigma		Alkyl Pi-Alkyl			H-Bond Donor Acceptor

**Table 5:** Interaction types, distances (Å) and amino acid residues in each molecular complex.

Complex	Residues	Distance (Å)	Interaction Type	ND24	Ala54	4.04	Alkyl
Acarbose	Glu211	3.71	Carbon Hydrogen Bond		Trp209	2.5	Conventional Hydrogen
	Arg345	1.81–2.69	Conventional Hydrogen		Trp209	4.28–5.09	Pi-Alkyl
	Arg69	1.95–2.85	Conventional Hydrogen		Trp362	4.71	Pi-Alkyl
	Asp70	2.77–2.93	Conventional Hydrogen		Tyr351	5.03	Pi-Alkyl
	Glu210	2.87–3.27	Conventional Hydrogen		Tyr63	4.39	Pi-Alkyl
	Glu211	3.02–3.11	Conventional Hydrogen		Glu210	3.28	Pi-Anion
	Gly140	1.96	Conventional Hydrogen		Glu211	3.65	Pi-Anion
	Leu208	2.62	Conventional Hydrogen		Glu456	4.14	Pi-Anion
	Trp209	3.32–3.47	Conventional Hydrogen		Tyr351	4.27–5.69	Pi-Pi Stacked
	Trp139	3.32–3.88	Pi-Donor Hydrogen Bond		Tyr351	5.26	Pi-Pi T-Shaped
	Trp67	3.78	Pi-Donor Hydrogen Bond		Trp67	3.48–3.76	Pi-Sigma
	Tyr351	3.36–3.88	Pi-Donor Hydrogen Bond	ND28	Arg345	2.59	Conventional Hydrogen
ND9	Glu210	2.02	Conventional Hydrogen		Glu211	2.69–2.83	Conventional Hydrogen
	Trp209	2.85	Conventional Hydrogen		Tyr351	2.96	Conventional Hydrogen
	Trp362	4.69	Pi-Alkyl		Tyr63	2.55	Conventional Hydrogen
	Trp473	4.85	Pi-Alkyl		Trp209	4.97	Pi-Alkyl
	Glu210	3.52	Pi-Anion		Trp362	4.96	Pi-Alkyl
	Glu456	3.87	Pi-Anion		Trp473	4.37	Pi-Alkyl
	Trp139	4.92–5.36	Pi-Pi Stacked		Glu210	3.69–4.51	Pi-Anion
	Tyr63	5.03	Pi-Pi Stacked		Glu211	3.72	Pi-Anion
	Trp139	4.85–5.34	Pi-Pi T-Shaped		Tyr351	4.02–5.85	Pi-Pi Stacked
ND8	Ala54	4.23	Alkyl		Trp139	5.66	Pi-Pi T-Shaped
	Trp209	2.48	Conventional Hydrogen		Trp362	5.42	Pi-Pi T-Shaped
	Trp209	4.46–5.25	Pi-Alkyl	ND33	Tyr351	2.95	Conventional Hydrogen
	Trp362	4.75	Pi-Alkyl		Trp209	2.61	Conventional Hydrogen
	Tyr351	4.95	Pi-Alkyl		Trp473	4.75	Pi-Alkyl
	Tyr63	4.39	Pi-Alkyl		Glu210	3.43	Pi-Anion
	Glu210	3.26	Pi-Anion		Glu211	3.84	Pi-Anion
	Glu211	3.65	Pi-Anion		Glu456	3.94	Pi-Anion
	Glu456	4.18	Pi-Anion		Tyr63	4.93	Pi-Pi Stacked
	Tyr351	4.30–5.61	Pi-Pi Stacked		Tyr351	4.36–5.88	Pi-Pi Stacked
	Tyr351	5.29	Pi-Pi T-Shaped		Tyr351	5.53	Pi-Pi T-Shaped
	Trp67	3.61–3.84	Pi-Sigma	ND37	Asp70	2.42	Conventional Hydrogen
ND14	Asp70	2.95	Conventional Hydrogen		Glu210	2.63–2.9	Conventional Hydrogen
	Glu210	3.09	Conventional Hydrogen		Glu211	2.96	Conventional Hydrogen
	Trp209	2.4	Conventional Hydrogen		Trp209	2.45	Conventional Hydrogen
	Glu210	3.58–3.60	Pi-Anion		Glu210	3.54	Pi-Anion
	Glu211	3.78	Pi-Anion		Glu211	3.86	Pi-Anion
	Glu456	4.15	Pi-Anion		Glu456	4.09	Pi-Anion
	Trp67	2.75	Pi-Donor Hydrogen Bond		Trp67	2.74	Pi-Donor Hydrogen Bond
	Tyr351	5.04–5.61	Pi-Pi Stacked		Tyr351	5.04–5.62	Pi-Pi Stacked
	Tyr63	5.09	Pi-Pi Stacked		Tyr63	5.05	Pi-Pi Stacked
	Trp139	4.83–5.20	Pi-Pi T-Shaped		Trp139	4.83–5.27	Pi-Pi T-Shaped
	Tyr351	5.17	Pi-Pi T-Shaped		Tyr351	5.27	Pi-Pi T-Shaped
	Colored background highlights amino acid residues shared between the docked ligands and acarbose, indicating conserved binding site interactions						

**Table 6:** Drug-likeness properties of the designed compounds.

	ND8	ND9	ND14	ND24	ND28	ND33	ND37
M.W	392.50	455.30	430.42	504.21	439.27	414.47	446.48
LogP	6.03	5.85	2.78	6.58	2.85	5.16	2.87
N. rot	2	2	2	2	2	2	2
N. Ha	4	6	10	6	10	6	10
N. Hd	1	1	7	3	3	1	5

M.W: Molecular weight (g mol^−1^); N. rot: Num. rotatable bonds; N. Ha: Num. H-bond acceptors; N. Hd: Num. H-bond donors.

**Table 7:** Pharmacokinetic and pharmacodynamic properties of the analyzed compounds.

Compounds	ND8	ND9	ND14	ND24	ND28	ND33	ND37
Caco2 permeability	−0.21	−0.03	−0.79	−0.50	−0.47	−0.04	−0.52
Intestinal absorption	89.8	100	83.92	97.55	89.78	100	74.74
Skin permeability	−2.73	−2.73	−2.73	−2.73	−2.73	−2.73	−2.73
VDss	−0.29	−0.11	0.10	0.075	−0.24	−0.11	0.08
BBB permeability	0.48	−0.37	−1.55	−1.29	−1.97	−0.06	−1.02
CNS permeability	−1.20	−1.37	−3.77	−1.38	−3.60	−1.46	−2.33
CYP2D6 substrate	No	No	No	No	No	No	No
CYP3A4 substrate	Yes	Yes	No	No	No	Yes	No
CYP1A2 inhibitor	Yes	Yes	No	Yes	No	Yes	Yes
CYP2C19 inhibitor	Yes	Yes	No	Yes	No	Yes	Yes
CYP2C9 inhibitor	Yes	Yes	No	Yes	No	Yes	No
CYP2D6 inhibitor	No	No	No	No	No	No	No
CYP3A4 inhibitor	Yes	Yes	Yes	Yes	Yes	Yes	Yes
Total clearance	0.58	0.49	0.75	0.44	0.59	0.51	0.82
Hepatotoxicity	No	No	No	No	Yes	No	Yes
Skin sensitisation	No	No	No	No	No	No	No

**Table 8:** MM/GBSA binding free energy (ΔG, kcal/mol) values of the selected ligand–2F6D complexes obtained after molecular dynamics simulation, showing the relative binding affinities of the investigated compounds compared with the reference inhibitor acarbose.

Complexes	MM/GBSA ΔG (kcal/mol)
**2F6D-ND08**	−58.06
**2F6D-ND33**	−41.41
**2F6D-ND37**	−51.49
**2F6D-Acarbose**	−74.35

**Table 9:** The formulas and threshold values of the calculated parameters, which are used to prove the validation of the developed model.

Parameter	Equation	Threshold
Coefficient of determination R^2^	$1-\frac{\sum_{i=1}^{n}{{(y}_{obseved}^{i}-y_{predicted}^{i})}^{2}}{\sum_{i=1}^{n}{\left(y_{observed}^{i}-\bar{y}\right)}^{2}}$	≥0.6
Adjusted coefficient of determination R^2^_adj_	$1-(\frac{\left(1-R^{2})(n-1\right)}{n-p-1})$	≥0.6
Cross-validation coefficient Q^2^	$1-\frac{\sum_{i=1}^{n}{\left(y_{observed}^{i}-y_{predicted}^{i}\right)}^{2}}{\sum_{i=1}^{n}{\left(y_{observed}^{i}-\bar{y}\right)}^{2}}$	≥0.5
Test set coefficient of determination R^2^_ext_	$1-\frac{\sum_{i=1}^{n}{{(y}_{obseved}^{i}-y_{predicted}^{i})}^{2}}{\sum_{i=1}^{n}{\left(y_{observed}^{i}-\bar{y}\right)}^{2}}$	≥0.6
Root mean square error RMSE	$\sqrt{\frac{1}{n}\sum_{i=1}^{n}{\left(y_{predicted}^{i}-y_{observed}^{i}\right)}^{2}}$	≈0
Variance inflation factor VIF	$\frac{1}{1-R_{j}^{2}}$	0 < VIF < 5

## Data Availability

The original contributions presented in this study are included in the article/Supplementary Material. Further inquiries can be directed to the corresponding author.

## Supplementary Materials

The following supporting information can be downloaded at: https://www.mdpi.com/article/10.3390/ijms27135696/s1.
