# Supplementary material for: In Silico Design of Pyrimidine Derivatives as Potential α-Glucosidase Inhibitors: QSAR, Molecular Docking, ADMET, and Molecular Dynamics Studies
Source: Int J Mol Sci. 2026 Jun 24;27(13):5696. doi: 10.3390/ijms27135696 (PMC13361322; doi:10.3390/ijms27135696)
Supplement: Supplementary file 1 [file ijms-27-05696-s001.zip › ijms-4215732-supplementary.pdf]

In silico design of pyrimidine derivatives as potential α-glucosidase inhibitors: QSAR, molecular docking, ADMET, and molecular dynamics studies

Oussama Abchir[1], Bouchra Rossafi [1], Amal Bouribab[1], Bouchra Es-Sounni [2], Rodouan Touti [3], Imane Yamari [1], Abdelouahid Samadi [4,*], Samir Chtita[1*]

Supplementary materials

Table S1. Dataset of compounds (3a–3ag) showing experimental antidiabetic activity (pIC_{50}) alongside selected molecular descriptors, including frontier orbital energy (E_{LUMO+1}), number of symmetric atoms, and topological autocorrelation indices (ATSC5e and GATS3p) used for QSAR modeling

Variables	pIC_{50}	$ELUMO_{+1}$	Symmetric atoms	ATSC5e	GATS3p
3a	4.26922	-0.83	4	-0.39	0.95954
3b	4.42251	-0.74	4	-0.24	1.01934
3c	4.06905	-0.62	4	-0.36	0.96025
3d	4.57349	-1.09	4	-0.54	0.97638
3e	3.52724	-1.11	2	-0.30	0.91034
3f	3.71265	-1.12	2	-0.29	0.97638
3g	4.03953	-1.00	2	-0.33	0.99361
3h	4.37059	-0.96	4	-0.54	0.97638
3i	4.43533	-0.86	4	-0.36	1.02775
3j	3.94195	-0.71	4	-0.41	0.97111
3k	4.78516	-1.22	4	-0.64	1.00201
3l	4.55284	-1.09	2	-0.49	1.02214
3m	4.20273	-0.96	4	-0.50	0.96858
3n	4.31515	-0.87	4	-0.33	1.01044
3o	4.12263	-0.75	4	-0.36	0.96025
3p	4.18442	-0.67	4	-0.20	1.02005
3q	3.91116	-0.58	4	-0.23	0.96530
3r	3.89143	-0.96	2	-0.27	0.99233
3s	3.79588	-0.77	2	-0.33	0.99361
3t	3.72469	-0.67	2	-0.15	1.04044
3u	3.65208	-0.60	2	-0.26	0.99233
3w	3.86646	-1.07	2	-0.48	1.02214
3x	3.59397	-1.01	0	-0.30	1.05535
3y	3.43133	-1.02	4	0.09	0.90637
3z	3.90798	-1.13	4	-0.02	0.94327
3aa	4.19044	-1.27	4	-0.02	0.94327
3ab	3.85078	-1.24	4	0.25	0.98375
3ac	3.64936	-1.20	4	0.28	0.92782
3ad	4.31515	-1.39	4	-0.07	0.98798
3ae	4.13727	-1.23	2	0.01	1.01939
3af	4.06854	-1.28	4	0.01	0.95103
3ag	3.98885	-1.25	4	0.26	0.98311

Table S2. QSAR model validation and Y-randomization results. The original model shows strong performance ($R = 0.9135$, $R^2 = 0.8345$, $Q^2 = 0.7583$), while all random models give low or negative Q^2 values, confirming the model is not due to chance.

Model	R	R^2	Q^2
**Original**	**0.913492827**	**0.834469145**	**0.758329802**
**Random 1**	0.51024273	0.260347644	-0.15424427
**Random 2**	0.32078661	0.102904049	-0.380515525
**Random 3**	0.404000919	0.163216743	-0.311489758
**Random 4**	0.486158359	0.23634995	-0.080852732
**Random 5**	0.190032883	0.036112497	-0.453161594
**Random 6**	0.151613598	0.022986683	-0.570761609
**Random 7**	0.503521686	0.253534089	-0.145107854
**Random 8**	0.304594242	0.092777653	-0.442812289
**Random 9**	0.507887978	0.257950198	-0.109778007
**Random 10**	0.340031076	0.115621133	-0.449295657
**Random 11**	0.401552186	0.161244158	-0.242839228
**Random 12**	0.640179961	0.409830382	0.086465711
**Random 13**	0.156427382	0.024469526	-0.437816603
**Random 14**	0.673692467	0.45386154	0.179362242
**Random 15**	0.407427992	0.165997569	-0.200802455
**Random 16**	0.496938699	0.24694807	-0.096397945
**Random 17**	0.521006754	0.271448037	-0.172755052
**Random 18**	0.272338484	0.07416825	-0.423731105
**Random 19**	0.379391205	0.143937687	-0.338835452
**Random 20**	0.40348762	0.162802259	-0.210569436
**Random 21**	0.343768845	0.118177019	-0.248599834
**Random 22**	0.314040785	0.098621614	-0.489532022
**Random 23**	0.340917593	0.116224805	-0.371230355
**Random 24**	0.527378949	0.278128556	-0.093508939
**Random 25**	0.271490919	0.073707319	-0.356441048
**Random 26**	0.115710666	0.013388958	-0.43108666
**Random 27**	0.418499783	0.175142068	-0.102357227
**Random 28**	0.223862912	0.050114603	-0.414587782
**Random 29**	0.43952661	0.193183641	-0.159545491
**Random 30**	0.450579787	0.203022145	-0.275852173
**Random 31**	0.363530054	0.1321541	-0.357150135
**Random 32**	0.301998258	0.091202948	-0.522238717
**Random 33**	0.311686102	0.097148226	-0.454572524
**Random 34**	0.377471732	0.142484908	-0.522269282
**Random 35**	0.420273489	0.176629805	-0.37578685
**Random 36**	0.223422805	0.04991775	-0.420934994
**Random 37**	0.544904671	0.296921101	-0.049095564
**Random 38**	0.346769412	0.120249025	-0.448494432
**Random 39**	0.232970264	0.054275144	-0.458511033
**Random 40**	0.282000739	0.079524417	-0.440537272
**Random 41**	0.381885753	0.145836729	-0.344092892
**Random 42**	0.40158253	0.161268528	-0.254959339
**Random 43**	0.555515677	0.308597667	0.031041876

**Random 44**	0.455775087	0.20773093	-0.132855119
**Random 45**	0.514437708	0.264646156	-0.168374507
**Random 46**	0.51860773	0.268953978	-0.166431847
**Random 47**	0.209918817	0.04406591	-0.448057936
**Random 48**	0.416973358	0.173866781	-0.213960495
**Random 49**	0.376771014	0.141956397	-0.26769842
**Random 50**	0.291137004	0.084760755	-0.520995366
**Random 51**	0.534180314	0.285348608	-0.093396339
**Random 52**	0.571040388	0.326087124	0.007729386
**Random 53**	0.231205437	0.053455954	-0.505628779
**Random 54**	0.486581354	0.236761414	-0.143571487
**Random 55**	0.203436051	0.041386227	-0.350128559
**Random 56**	0.244409573	0.05973604	-0.574105397
**Random 57**	0.335744576	0.11272442	-0.375044525
**Random 58**	0.514786929	0.265005582	-0.1858654
**Random 59**	0.210602019	0.04435321	-0.499621502
**Random 60**	0.471304653	0.222128076	-0.203704741
**Random 61**	0.244806041	0.059929998	-0.73235693
**Random 62**	0.300440545	0.090264521	-0.370160603
**Random 63**	0.379868786	0.144300295	-0.294902103
**Random 64**	0.550385605	0.302924314	-0.003404823
**Random 65**	0.336838129	0.113459925	-0.288179258
**Random 66**	0.25932208	0.067247941	-0.482171853
**Random 67**	0.482447723	0.232755806	-0.14212866
**Random 68**	0.398883425	0.159107987	-0.208020185
**Random 69**	0.322268127	0.103856745	-0.422443789
**Random 70**	0.307392244	0.094489992	-0.602906347
**Random 71**	0.265372085	0.070422343	-0.416546573
**Random 72**	0.473172804	0.223892503	-0.26773224
**Random 73**	0.298070117	0.088845795	-0.466724281
**Random 74**	0.291840371	0.085170802	-0.403432539
**Random 75**	0.495533217	0.245553169	-0.091414183
**Random 76**	0.293835068	0.086339047	-0.295810909
**Random 77**	0.373338134	0.139381363	-0.232413253
**Random 78**	0.389932352	0.15204724	-0.396754247
**Random 79**	0.300510418	0.090306511	-0.314927251
**Random 80**	0.337700714	0.114041772	-0.468094927
**Random 81**	0.376250227	0.141564234	-0.363490847
**Random 82**	0.291863235	0.085184148	-0.571024465
**Random 83**	0.263335833	0.069345761	-0.466400146
**Random 84**	0.55792778	0.311283408	-0.079792953
**Random 85**	0.441233559	0.194687054	-0.198103736
**Random 86**	0.516695177	0.266973906	-0.075741648
**Random 87**	0.17646985	0.031141608	-0.520555131
**Random 88**	0.410605026	0.168596488	-0.202309578
**Random 89**	0.407468626	0.166030681	-0.242110464
**Random 90**	0.273997364	0.075074556	-0.378839384
**Random 91**	0.260077299	0.067640201	-0.517398872
**Random 92**	0.229565774	0.052700444	-0.415780409

**Random 93**	0.395306203	0.156266994	-0.223442142
**Random 94**	0.289320769	0.083706508	-0.699634797
**Random 95**	0.51178243	0.261921255	-0.218267431
**Random 96**	0.247002344	0.061010158	-0.40081776
**Random 97**	0.298048835	0.088833108	-0.576091544
**Random 98**	0.244864758	0.05995875	-0.487551017
**Random 99**	0.446992392	0.199802199	-0.300641127
**Random 100**	0.383304291	0.14692218	-0.283553899
**Random Models Parameters**			
**Average R**	0.369720829	**Average Q^2**	-0.314800646
**Average R^2**	0.150183785	**cR_p^2**	0.763067659
